# Ag nanoparticles-decorated ZnO nanorod array on a mechanical flexible substrate with enhanced optical and antimicrobial properties

**DOI:** 10.1186/s11671-014-0712-3

**Published:** 2015-03-01

**Authors:** Yi Chen, Wai Hei Tse, Longyan Chen, Jin Zhang

**Affiliations:** Department of Chemical & Biochemical Engineering, University of Western Ontario, 1151 Richmond Street, London, Ontario Canada N6A 5B9; Department of Medical Biophysics, University of Western Ontario, 1151 Richmond Street, London, Ontario Canada N6A 5C1

**Keywords:** Nanorod array, Nanoparticles, Flexible substrate, Photoluminescence, Antimicrobial efficiency

## Abstract

Heteronanostructured zinc oxide nanorod (ZnO NR) array are vertically grown on polydimethylsiloxane (PDMS) through a hydrothermal method followed by an *in situ* deposition of silver nanoparticles (Ag NPs) through a photoreduction process. The Ag-ZnO heterostructured nanorods on PDMS are measured with an average diameter of 160 nm and an average length of 2 μm. ZnO NRs measured by high-resolution transmission electron microscope (HRTEM) shows highly crystalline with a lattice fringe of 0.255 nm, which corresponds to the (0002) planes in ZnO crystal lattice. The average diameter of the Ag NPs *in situ* deposited on the ZnO NRs is estimated at 22 ± 2 nm. As compared to the bare ZnO NRs, the heterostructured Ag-ZnO nanorod array shows enhanced ultraviolet (UV) absorption at 440 nm, and significant emission in the visible region (λ_em_ = 542 nm). In addition, the antimicrobial efficiency of Ag-ZnO heterostructured nanorod array shows obvious improvement as compared to bare ZnO nanorod array. The cytotoxicity of ZnO nanorod array with and without Ag NPs was studied by using 3 T3 mouse fibroblast cell line. No significant toxic effect is imposed on the cells.

**Electronic supplementary material:**

The online version of this article (doi:10.1186/s11671-014-0712-3) contains supplementary material, which is available to authorized users.

## Background

One-dimensional ZnO nanostructures including nanorods and nanowires have been intensively studied because of its wide band-gap, large shape anisotropy, and less light scattering capability [1-3]. To date, most reported techniques for producing ZnO nanorod (NR) arrays require expensive vacuum system, an/or high-temperature process, including chemical vapor deposition [4], thermal decomposition of precursors [5], oxidation of zinc metal, *etc*. [6,7]. Owing to high demand, huge efforts have been focusing on the development of biocompatible and mechanically flexible photovoltaic devices for their potentials in wearable devices and optical prosthetic devices [8,9]. It is, therefore, important to develop cost-efficient and low-temperature processes to deposit ZnO NRs on polymer substrates.

In addition, it is noted that the polydimethylsiloxane (PDMS) has been extensively used as the substrate in medical devices. However, the bacterial biofilm grown on PDMS limits its application *in vitro* and *in vivo* [10,11]. Silver nanoparticles (Ag NPs) have shown the enhanced surface plasmon resonance and demonstrated antimicrobial properties against both gram-negative and gram-positive bacteria [12-14]. Moreover, enhanced optical absorption and photoelectronic current have been observed in ZnO nanostructures doped with noble metallic nanostructures because the noble metals have lower Fermi energy level, and promote the interfacial electron transfer process [15-17]. However, most reported studies focus on decorating metallic nanostructures on zinc oxide (ZnO) nanowire, or nanotubes to obtain enhanced properties, e.g., Raman scattering, photocatalytic activity, *etc*. [18-20]. Very few studies have been reported on the deposition of biocompatible and mechanically flexible hybrid ZnO NR array on polymer substrates, such as PDMS, to have both high photon harvest and inhibition of the growth of bacteria. The major challenges lie in growing ZnO NR array on a polymer substrate, and maintain well-controlled surface of ZnO NRs for self-nucleation of the additional Ag nanoparticles.

Here, we have developed a two-step, cost-effective process. First, a hydrothermal method is applied in producing ZnO NRs vertically grew on PDMS at low temperature; a photoreduction process is developed for *in situ* reducing and depositing Ag NPs on ZnO NRs. The prepared heteronanostructures Ag-ZnO has been carefully characterized by X-ray diffraction (XRD), field emission scanning electron microscopy (FE-SEM), transmission electron microscopy (TEM), and X-ray photoelectron spectroscopy (XPS). The surface plasmon resonance and photoluminescence of the heterostructured nanorod array have been studied by UV-vis and fluorospectrometer, respectively. In addition, hybrid Ag-ZnO nanorod array was treated by gram-negative and gram-positive bacteria in this paper to evaluate the antimicrobial efficiency of the hybrid nanostructures. We expect that the heteronanostructured Ag-ZnO rods deposited on the polymer substrate with flexible mechanic properties could be applied in wearable devices and/or optical prosthetic devices.

## Methods

### Fabrication of heterostructured nanorods on PDMS

First, ZnO nanorod array was grown on PDMS substrate by a modified low-temperature hydrothermal method [19,20]. PDMS substrate was dipped in 0.01 M zinc acetate dehydrate aqueous solution (Zn(CH_3_COO)_2_ · 2H_2_O, 99.999%; Sigma-Aldrich, St. Louis, MO, USA) several times following the heat treatment at 100°C for 1 h to obtain a dense seed ZnO layer on PDMS substrate. ZnO seeds coated PDMS film was then immersed into the mixture of zinc nitrate hexahydrate (Zn(NO_3_)_2_ · 6H_2_O, 98%, 0.025 M, Sigma-Aldrich) and haxamethylenetetramine ((CH_3_)_6_ N_4_, HMTA 99%, 0.025 M, Sigma-Aldrich) to form ZnO nanorod array vertically grown on PDMS. After heating at 95°C for 3 h, the products were rinsed with distilled water and acetone. The ZnO nanorod array deposited on PDMS were dried in an oven at 70°C for 1 h.

To modify the surface of ZnO NRs with Ag NPs, an *in situ* coating method is developed. Silver nitrate (AgNO_3_, 99%, 10 mM, Sigma-Aldrich) was dissolved in a solution with 10 ml distilled and deionized (DD) water and 0.5 ml ethanol [21,22]. The mixture was stirred at room temperature until a clear solution is formed. Following that, the ZnO-coated PDMS was merged into the AgNO_3_ solution, which was exposed under a ultravoilet (UV) reactor (1 kW Hg(Xe) with λ = 220 to 260 nm; Luzchem, Gloucester, ON, Canada) for 10 min at room temperature.

**Figure 1:**
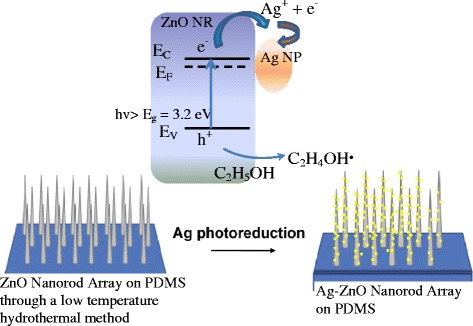
***In situ*** **process for depositing Ag NPs on ZnO nanorods which grew on a PDMS substrate.**

As shown in Figure 1, the ZnO NR grown on PDMS is exposed under a UV light (*hv*) which is larger than the band gap of ZnO (*E*_g_ = 3.2 eV), leading to electron-hole pairs. At the present of C_2_H_5_OH, the holes (h^+^) are consumed to produce ethoxy radicals C_2_H_4_OH. Meanwhile, the accumulated electrons (e^−^) contribute to reduce AgNO_3_ to form Ag nanoparticles *in situ* on the surface of ZnO nanorods [23,24]. The final products were resined by ethanol and DI water three times and dried in a vacuum oven.

### Materials characterization

Microstructures of the hybrid nanorod array was investigated by a field emission scanning electron microscope (LEO 1530, 3 kV; Zeiss, Jena, Germany), a transmittance electron microscope (Philips CM10, 80 kV; Philips, Amsterdam, The Netherlands), and a high-resolution transmittance electron microscope (HRTEM, JEOL 2010 FEG, 200 kV; JEOL, Akishima-shi, Japan). The elemental analysis of the hybrid nanorod was carried out by the energy dispersive X-ray spectroscopy (EDX) combined with FE-SEM. Meanwhile, a X-ray powder diffraction (Rigaku Miniflex II Instrument; Rigaku, Shibuya-ku, Japan) using nickel-filter Cu Kα radiation (λ = 1.5406 Ǻ) was used to investigate the crystal structures of the hybrid nanorod array. The UV-visible absorption spectra and room temperature photoluminescence (PL) spectra were obtained through the measurements of a UV-vis-NIR spectrophotometer (UV-3600, Shimadzu, Kyoto, Japan) and a spectrofluorometer (QuantaMster™30, PTI), respectively. In addition, a X-ray photoelectron spectroscopy (AXIS Ultra, Kratos, Chestnut Ridge, NY, USA) was used to further analyze the chemical components of heterostructured Ag-ZnO nanorods.

### Antimicrobial assay

The antibacterial capability of the Ag NP-decorated ZnO nanorod arrays (Ag-ZnO) was assessed by a drop-test approach on gram-positive bacterium, *Staphylococcus aureus* (ATCC) and gram-negative bacteria, *Escherichia coli* (*E. coli* BL21, ATCC), respectively [25,26]. The gram-negative bacteria, *E. coli*, was used as an example to briefly indicate the test. *E. coli* was cultured overnight at LB broth at 37°C until a density of 10^8^ CFU/ml was approached. The culture was then diluted to 10^6^ CFU/ml with sterile phosphate-buffered saline (PBS). Then 100 μL of the above PBS diluted bacteria suspension was then placed onto the surface of samples. The samples were stored at the ambient room temperature for a period of time interval (1, 2, 4, 12 h). The surface of the sample cultured with bacteria at each time period was washed by 5 ml of PBS to remove the bacterial residue on the samples into the PBS. Then 10 μl of each of the bacteria suspensions was placed on the LB agar. The number of bacteria that survived on the petri-dish was then counted after incubation for 24 h at 37°C. All experiments were run in triplicate.

### Cytotoxicity

Fifty thousand 3 T3 mouse fibroblast cells were seeded onto a 24-cell culture plate and incubated in a 5% CO_2_ incubator overnight, and samples of Ag-ZnO nanorods were incubated with cells for 24 h per well. Different amount of hybrid nanostructures (0.03, 0.07, 0.10 mg/ml) were used in the test. The control sample was cultured cells without the produced heteronanostructured samples. The MultiTox-Fluor Multiplex Cytotoxicity Assay Kit (Promega, Sunnyvale, CA, USA) is used to measure relative cell viability. The measurement process followed the protocol of the product [27]. The reagent was added to the 96-well plate and incubated at 37°C for 30 min. Triplicates of all samples were measured. Fluorescent signals were measured at an excitation of 400 nm and an emission of 505 nm for live cells, then at an excitation (λ_ex_) of 485 nm and an emission (λ_em_) of 520 nm for dead cells.

## Results and discussion

### Materials characterization

**Figure 2:**
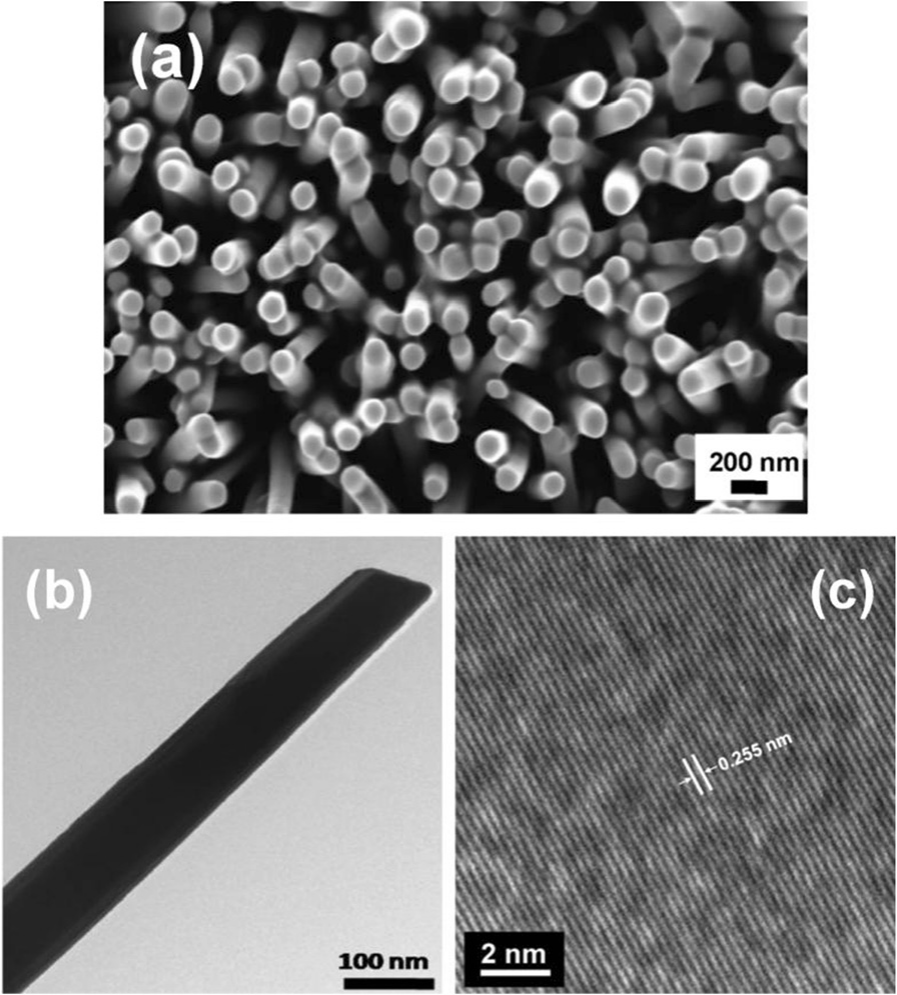
**(a) FE-SEM micrograph of aligned ZnO nanorod array on PDMS, (b) TEM micrograph of ZnO nanorod, and (c) HRTEM micrograph of the ZnO nanorod.**

The surface morphology and detailed internal structures of ZnO nanorods on PDMS have been studied by FE-SEM and TEM. Figure 2a shows the top-view of the FE-SEM micrograph of the ZnO nanorod array vertically and uniformly grown on the PDMS substrate. The average diameter of ZnO nanorods is estimated at 160 ± 5 nm with a length of 2 μm. The shape and size of the typical as-grown ZnO nanorod are revealed in the TEM micrograph (Figure 2b), which is consistent with the result of the FE-SEM micrograph. The HRTEM micrograph (Figure 2c) further indicates that the nanorods are highly crystalline with a lattice fringe of 0.255 nm, which corresponds to the (0002) planes in the ZnO crystal lattice.

**Figure 3:**
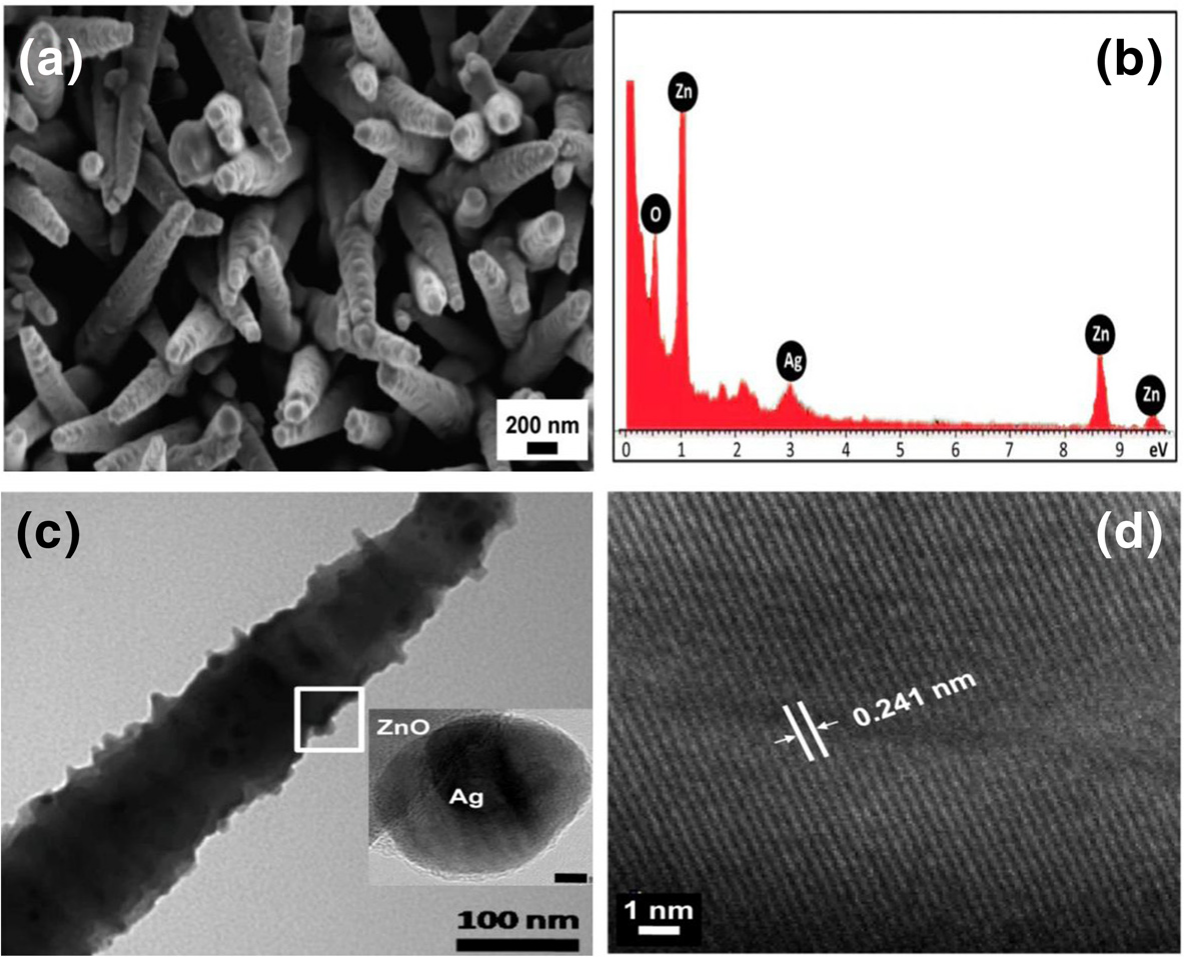
**Morphologies and structures of the hybrid nanostructures. (a)** FE-SEM micrograph of Ag-ZnO nanorod array on PDMS, **(b)** EDX spectrum of Ag-ZnO nanorods, **(c)** TEM micrograph of Ag-ZnO nanorod, inset small image is the HRTEM micrograph of Ag-ZnO interface with 5 nm scan bar, **(d)** HRTEM micrograph of Ag NP lattice fringes.

The heterostructure of Ag-ZnO nanorods is clearly shown in Figure 3. Compared to bare ZnO NRs, the nanostructured Ag-ZnO NRs are not smooth, but decorated with dots as shown in Figure 3a. The Ag-ZnO heterostructured nanorods were measured by EDX. Figure 3b indicate the three elements are Zn, O, and Ag (1.66 ± 0.5 at. %). It indicates that the large amount of spherical Ag NPs is *in situ* generated on the ZnO NRs surface. Furthermore, Ag-ZnO NRs were studied by HRTEM. The average diameter of spherical Ag nanoparticles is estimated at 10 ± 5 nm as shown in Figure 3c. The small-inset image shows a HRTEM micrograph of Ag-ZnO NRs. In addition, the interplanar distance is measured at 0.241 nm as shown in Figure 3d which corresponds to the d-spacing of the [111] crystal plane of Ag NPs. The interface between ZnO NR and Ag NP is highlighted by the white rectangle.

**Figure 4:**
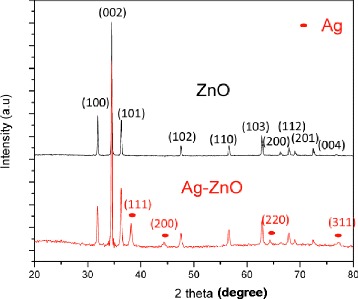
**XRD patterns of pristine ZnO nanorods and Ag-ZnO nanorods.**

The XRD patterns of the ZnO and Ag-ZnO nanorods are displayed in Figure 4. The diffraction peaks of ZnO NRs array match with that of typical hexagonal wurzite structure of ZnO (JCPDS card no. 36-1451). The new peaks at the positions of 2*θ* = 38.12°, 44.28°, 64.25°, and 77.47° that are attributed to (111), (200), (220), and (311) crystalline planes of Ag crystal structure (JCPDS card no. 04-0783) were clearly found from the XRD profile of Ag-ZnO heterostructured nanorods. In addition, there is no remarkable shift and intensity change to all diffraction peaks, implying that no Zn_1-x_Ag_x_O was formed. The Ag NPs particle size can be estimated by Scherrer’s equation based on the XRD pattern [28]:

1 $$ K=\frac{K\ \lambda }{\beta\ \cos\ \theta } $$

where *D* is the Ag NPs particle size, *K* is the constant on crystallite shape (0.89), λ is the X-ray wavelength, β is the full width at half max (FWHM) of Ag (111) diffraction peak, and θ is the Bragg angle. The average particle size of Ag NPs deposited on ZnO nanorods is around 22.9 nm which is consistent with the result of TEM.

**Figure 5:**
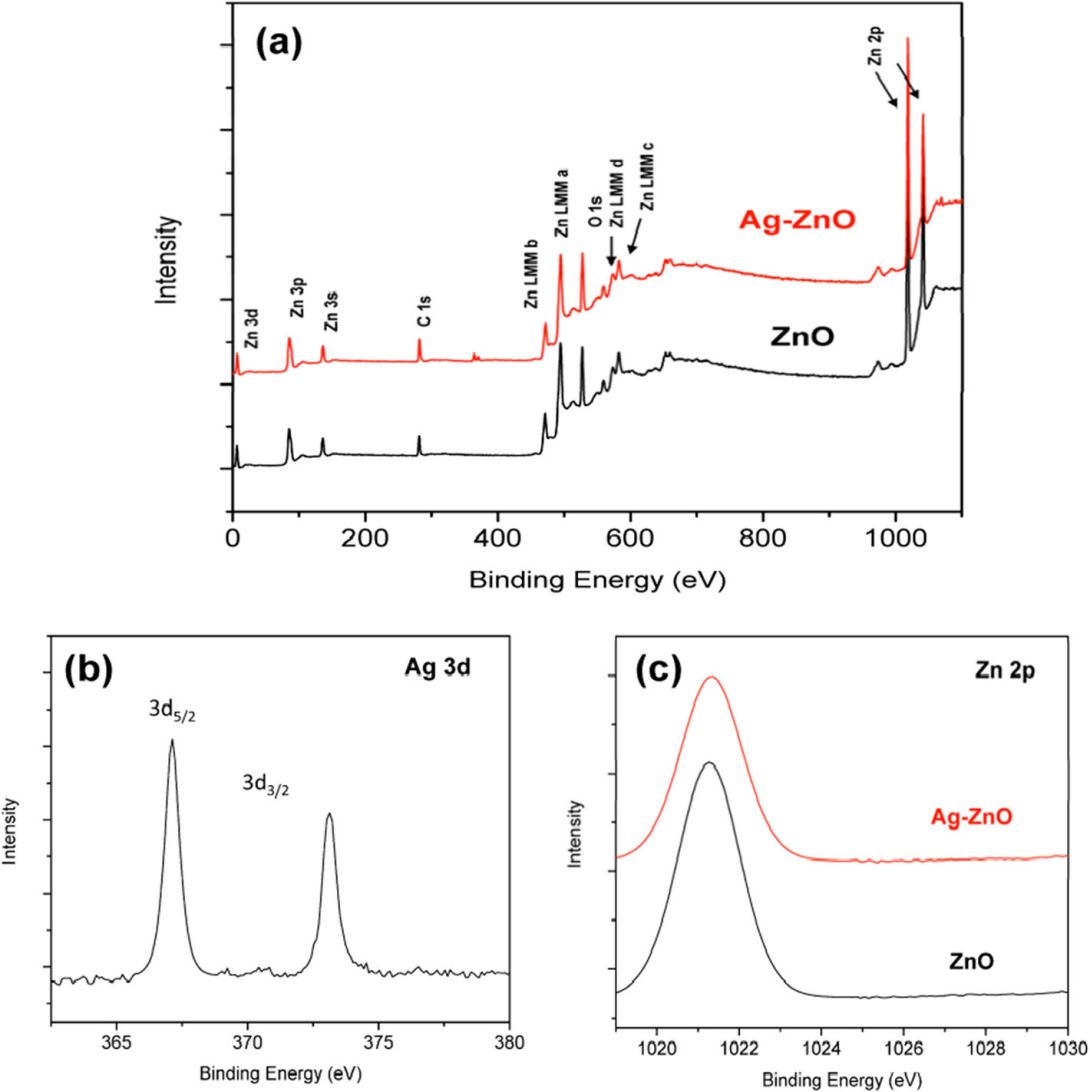
**XPS spectra of samples. (a)** Full profile XPS spectra of bare ZnO nanorod array and Ag-ZnO heterostructured nanorod arrays, high-resolution XPS bare ZnO nanorods, and Ag-ZnO heterostructured nanorods; **(b)** Ag 2d and **(c)** Zn 2p.

As a supplementary to XRD pattern, Figure 5a shows the full scan XPS spectra of as-growth ZnO NRs and Ag-ZnO NRs. There are only C, Zn, and O element peaks. An additional Ag peak is observed for heterostructured Ag-ZnO nanorods. The presence of C might be caused by the XPS instrument. High-resolution spectrum of Ag 3d to Ag-ZnO nanorods As shown in Figure 5b shows Ag 2d_3/2_ and Ag 2d_5/2_ peaks located at 367.12 and 373.2 eV, which indicates that Ag NPs deposited on ZnO nanorods are neither oxidized silver nor ionic species. Furthermore, the binding energies of Ag 3d for Ag-ZnO shift to the lower binding energy compared with bare Ag metal NPs (368.2 and 374.2 eV for Ag 3d_5/2_ and Ag 3d_3/2_, respectively) should be caused by the interaction between ZnO NRs and Ag NPs.

When Ag NPs are *in situ* deposited onto ZnO NRs, the positions of their corresponding Fermi energy levels need to adjust to the same value. Therefore, there are some free electrons above the new Fermi level of Ag NPs, which could return to the conduction band of ZnO NRs, leading to high valence of Ag NPs [22-24]. Since the binding energy of monovalent Ag is much lower than zero-valent of Ag, the binding energy of Ag 3d shifts to the lower binding energy. The position of Zn 2p_3/2_ for Ag-ZnO heterostructured nanorods (1021.33 eV) and bare ZnO nanorods (1021.36 eV) are almost the same. This result also confirmed that Zn^2+^ ions form in the bare ZnO nanorods and Ag-ZnO heterostructured nanorods.

### Optical properties

**Figure 6:**
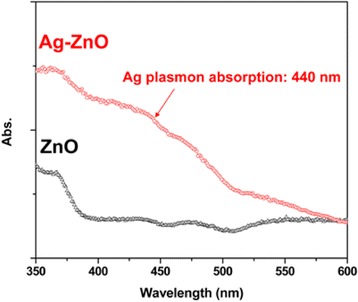
**UV-visible spectra of ZnO and Ag-ZnO nanorod arrays on PDMS.**

Both optical absorption and luminescent emission of the heterostructured Ag-ZnO nanorods on PDMS were studied. Figure 6 shows the UV-visible absorption spectra of ZnO nanorod array on PDMS with and without the deposition of Ag NPs. The bare ZnO nanorod array on PDMS exhibits the typical UV absorption peak at 377 nm [1-3]. Whereas a new broad absorption band centering at 440 nm is observed to the Ag-ZnO nanorod array on PDMS. We also developed pure Ag NPs with 20 ± 5 nm in diameter deposited on PDMS. The surface plasmon resonance (SPR) of the pure Ag NPs on PDMS is around 430 nm (Additional file 1). The SPR shift can be explained by the following equation [29]:

2 $$ {\lambda}_p{\left[4{\pi}^2{c}^2{m}_{eff}{\varepsilon}_0/N{e}^2\right]}^{1/2} $$

where λ_p_ is the SPR wavelength of Ag NPs, m_eff_ is the effective mass of the free electron of Ag NP, and *N* is the electron density of Ag. It is noted that the free electrons of Ag NPs may transfer into ZnO NR in the process of *in situ* deposition to lead to the equal Fermi level of Ag NP and Zn NR [20,23]. This transfer results in the decrease of the Ag electron density (*N*) and, therefore, causes the red shift of the SPR wavelength of heterostructured Ag-ZnO NRs.

**Figure 7:**
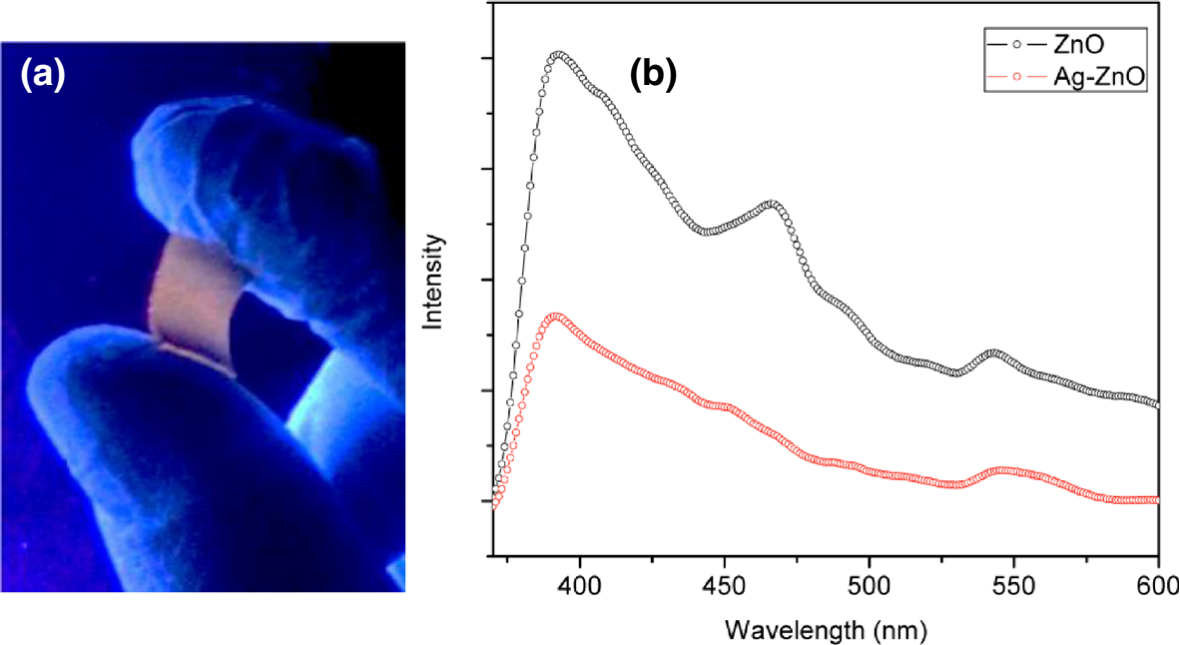
**Luminescence of the hybrid nanostructures. (a)** A photo of the flexible Ag-ZnO nanorod array on PDMS taken under a UV lamp (120 V/60 Hz Fisher). **(b)** Photoluminescent spectra of ZnO and Ag-ZnO heterostructured nanorod arrays on PDMS.

Figure 7a is a photo of the luminescent Ag-ZnO nanorod array deposited on the flexible PDMS substrate taken under a low-intensity UV lamp. The PL of as-growth ZnO NR array and Ag-ZnO NR array on PDMS were further measured at room temperature when the excitation wavelength (λ_ex_) is 325 nm. In Figure 7b, both two spectra show typical emission bands (λ_em_) of ZnO. The near UV emission around 390 nm corresponding to the near band edge (NBE) emission of ZnO, a blue emission located at 466 nm, and a broad green emission peak centering at around 542 nm which is related to oxygen vacancies, zinc vacancies, oxygen interstitials, and zinc interstitials [30]. The PL intensity of ZnO emission peaks decreases with Ag decoration in the ultraviolet region, resulting from the decreasing effect of electron-hole recombination in the hybrid Ag-ZnO nanorods where Ag NPs act as electron sinks [30,31].

### Antimicrobial activity

**Figure 8:**
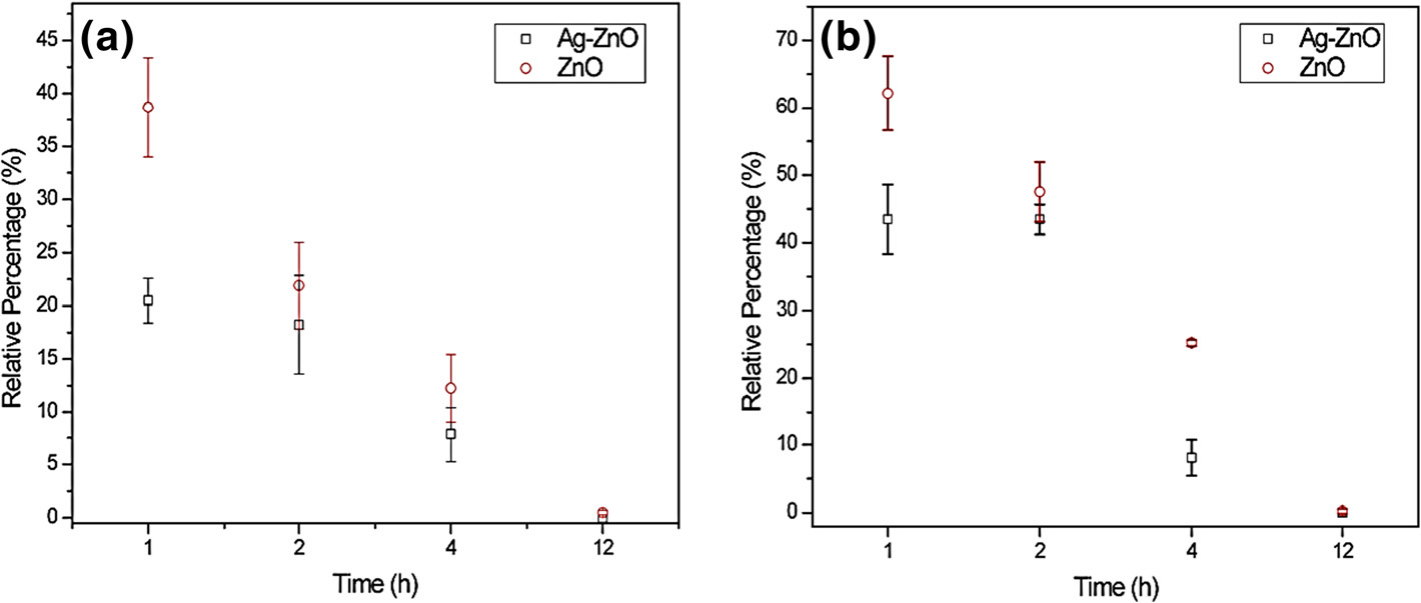
**Antimicrobial activities of ZnO and Ag-ZnO nanorod arrays on PDMS. (a)** Percentage survival of *S. aureus* vs. different incubation time; **(b)** Percentage survival of *E. coil* vs. different incubation time.

Different types of Ag-based and ZnO-based nanostructures have been studied before and have shown synergistic antimicrobial activity for both gram-positive and gram-negative bacteria [32]. The antimicrobial activities of bare ZnO and Ag-ZnO NR array on PDMS to both *S. aureus* and *E. coli* were evaluated quantitatively though the drop-test method, respectively. Figure 8 shows that Ag-ZnO NR array on PDMS is able to kill over 80% *S. aureus* and 55% *E. coli* when the culture time (*t*) is 1 h, respectively. The relative viability decrease to 5% in *S. aureus* and 15% in *E. coli* when *t* = 4 h. It shows that Ag-ZnO heterostructured nanorod array exhibits a higher antimicrobial efficiency (over 20%) on both gram-positive and gram-negative bacteria as compared to the bare ZnO NRs in the first-hour incubation. The relative viability percentage of gram-positive and gram-negative bacteria on both ZnO and Ag-ZnO NR array on PDMS is approaching to zero after an overnight incubation (*t* = 12 h). Ag NPs have demonstrated antimicrobial effect depending on a manner of superficial contact, where silver could inhibit enzymatic system of the respiratory chain, thereby influencing the DNA synthesis [32]. Meanwhile, the homogeneous formation and intensive distribution of small Ag NPs on ZnO nanorod arrays offering more chance for the bacterial cells contact with Ag NPs. The releasing of both Ag^+^ and Zn^2+^ from the nanomaterials with overtime incubation may also contribute to the increment of antimicrobial activity [32,33].

In addition, it is noted that both virgin ZnO NRs and Ag-ZnO NRs show higher antimicrobial activities to *S. aureus* than to *E. coli* at the first 2-h incubation. The results were consistent with previous report that the antimicrobial activity of ZnO is more effective for gram-positive than gram-negative bacteria as the former have simpler membrane structure [34].

### Cytotoxicity

**Figure 9:**
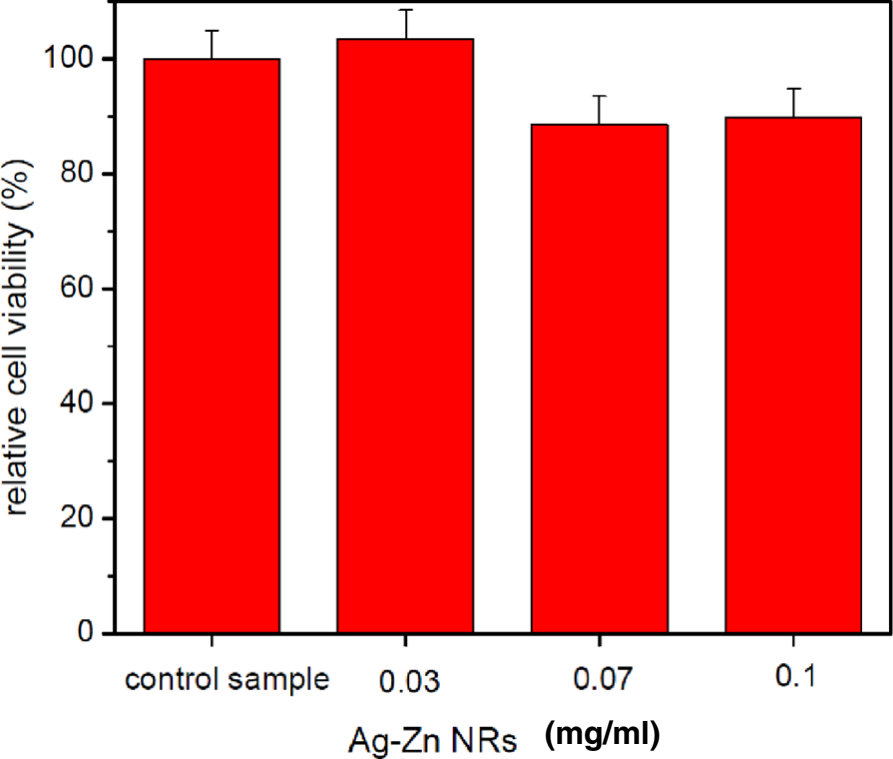
**Relative cell viability of control samples (no Ag-ZnO NRs) and Ag ZnO NRs.**

The cell response to heterostructured Ag-ZnO NRs was further investigated using NIH/3 T3 mouse fibroblast cell line. Control sample (no nanostructured materials) and Ag-ZnO NRs were incubated with cells for 24 h. Figure 9 shows the relative cell viability ([C_r_/C_o_] 100%) vs. different concentration of nanomaterials. The relative cell viabilities (%) of NIH/3 T3 mouse fibroblast cells treated by samples are correspondingly normalized to the control sample. Here, *C*_*o*_ is the viable cell numbers of the control sample, and *C*_*r*_ is the viable cell numbers treated with the heterostructured Ag-ZnO NRs. The error bars are the calculated standard deviation. The relative viability of cells treated with 0.03 mg/ml of Ag-ZnO NRs is about 103 ± 5%. The relative viabilities (%) of cells treated with higher concentrations of Ag-ZnO NRs (0.07 and 0.10 mg/ml) are higher than 88 ± 5% after 24-h incubation. The results indicate that no significant harmful effect is imposed to the cells.

## Conclusions

In summary, Ag-ZnO hybrid nanorod array has been deposited onto PDMS through a two-step chemical method, i.e., a hydrothermal method followed by a photoreduction process. The heterostructures of Ag NP-coated ZnO nanorod array (Ag-ZnO) have been thoroughly studied by SEM, TEM, XPS, UV-vis absorption spectrometry, and fluorescent spectrometry. The Ag-ZnO nanorods are uniformly deposited on PDMS with the diameter of 160 nm and the length of 2 μm. The average diameter of the Ag NPs in the heterostructures is estimated at 22 ± 2 nm. Our results indicate that the ZnO nanorods are highly crystalline with a lattice fringe of 0.255 nm, which corresponds to the (0002) planes in the ZnO crystal lattice. The UV absorption of ZnO nanorod array significantly increase after doping with Ag NPs, while the intensities of its emission in the UV region decrease as compared to that of bare ZnO nanorod array. In addition, the heterostructure of Ag-ZnO nanorod array on PDMS has been treated by both gram-positive bacteria, i.e., *S. aureus*, and gram-negative bacteria, i.e., *E. coli*. The antimicrobial effect of Ag-ZnO nanorod array shows obvious improvement at the early culture period as compared to bare ZnO nanorod array. The as-made Ag-ZnO nanorods have shown enhanced antimicrobial efficiency to gram-negative bacteria, *E. coli*, and gram-positive bacteria, *S. aureus*, as compared to the ZnO nanorods. The study of cytotoxicity indicates that the heterostructured Ag-ZnO NRs do not impose toxic effect on NIH/3 T3 mouse fibroblast cell line. The flexible Ag-ZnO nanorod array on PDMS shows well-tailored luminescent properties, superior antimicrobial efficiency, and good biocompatibility, therefore, it could be applied in wearable devices and optical prosthetic devices.

## Additional file

Additional file 1:
**UV-vis absorption of Ag NPs with 20 ± 5 nm in diameter deposited on PDMS through photoreduction process.**
