# Supplementary material for: Ag nanoparticles-decorated ZnO nanorod array on a mechanical flexible substrate with enhanced optical and antimicrobial properties
Source: Nanoscale Res Lett. 2015 Mar 1;10:106. doi: 10.1186/s11671-014-0712-3 (PMC4385129; doi:10.1186/s11671-014-0712-3)
Supplement: Additional file 1: — UV-vis absorption of Ag NPs with 20 ± 5 nm in diameter deposited on PDMS through photoreduction process. [file 11671_2014_712_MOESM1_ESM.pdf]

Supplementary document

Ag Nanoparticles-decorated ZnO Nanorod Array on A Mechanical Flexible Substrate with Enhanced Optical and Anti-microbial Properties

*Yi Chen, Wai Hei Tse, Longyan Chen, and Jin Zhang **

Department of Chemical & Biochemical Engineering, University of Western Ontario, London, Ontario, Canada, N6A 5B9
E-mail: jzhang@eng.uwo.ca

S1. Ag NPs with 20±5 nm in diameter deposited on PDMS through a photo-reduction process .

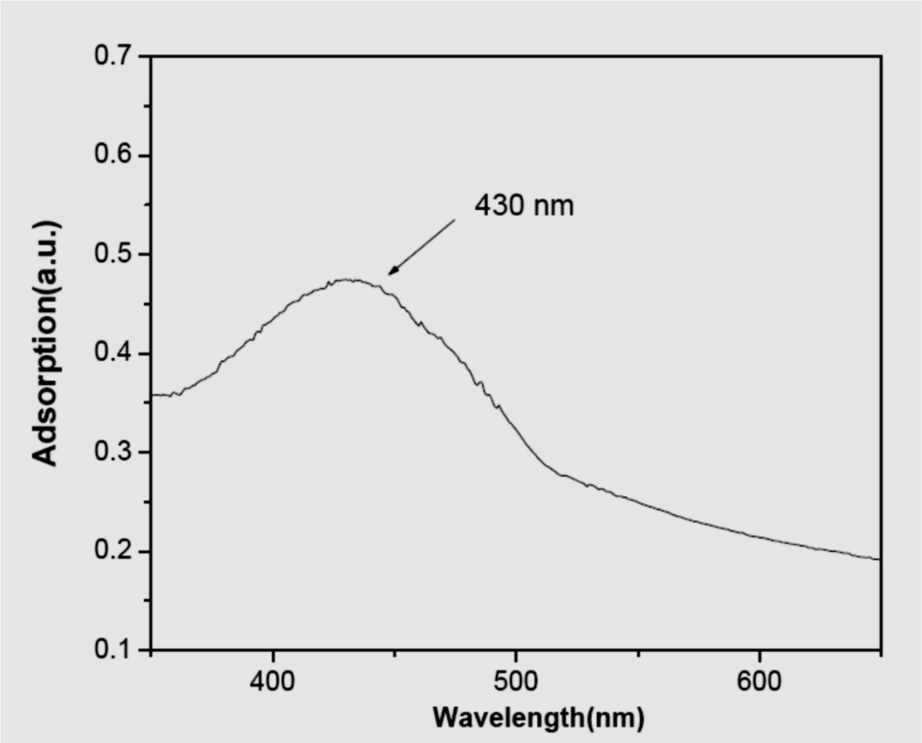

Figure S1. UV-vis absorption of Ag NPs with 22 nm in diameter deposited on PDMS through a photo-reduction process.
